# The Protective Effect of Glycyrrhetinic Acid on Carbon Tetrachloride-Induced Chronic Liver Fibrosis in Mice via Upregulation of Nrf2

**DOI:** 10.1371/journal.pone.0053662

**Published:** 2013-01-14

**Authors:** Shaoru Chen, Liyi Zou, Li Li, Tie Wu

**Affiliations:** The Pharmacy of GuangDong Medical College, DongGuan, GuangDong, China; University of South Florida College of Medicine, United States of America

## Abstract

This study was designed to investigate the potentially protective effects of glycyrrhetinic acid (GA) and the role of transcription factor nuclear factor-erythroid 2(NF-E2)-related factor 2 (Nrf2) signaling in the regulation of Carbon Tetrachloride (CCl_4_)-induced chronic liver fibrosis in mice. The potentially protective effects of GA on CCl_4_-induced chronic liver fibrosis in mice were depicted histologically and biochemically. Firstly, histopathological changes including regenerative nodules, inflammatory cell infiltration and fibrosis were induced by CCl_4_.Then, CCl_4_ administration caused a marked increase in the levels of serum aminotransferases (GOT, GPT), serum monoamine oxidase (MAO) and lipid peroxidation (MDA) as well as MAO in the mice liver homogenates. Also, decreased nuclear Nrf2 expression, mRNA levels of its target genes such as superoxide dismutase 3 (SOD3), catalase (CAT), glutathione peroxidase 2 (GPX2), and activity of cellular antioxidant enzymes were found after CCl_4_ exposure. All of these phenotypes were markedly reversed by the treatment of the mice with GA. In addition, GA exhibited the antioxidant effects in vitro by on FeCl_2_-ascorbate induced lipid peroxidation in mouse liver homogenates, and on DPPH scavenging activity. Taken together, these results suggested that GA can protect the liver from oxidative stress in mice, presumably through activating the nuclear translocation of Nrf2, enhancing the expression of its target genes and increasing the activity of the antioxidant enzymes. Therefore, GA may be an effective hepatoprotective agent and viable candidate for treating liver fibrosis and other oxidative stress-related diseases.

## Introduction

In spite of tremendous scientific advancement in the field of hepatology in recent years, liver problems are on the rise and account for a high death rate [1]. Hepatic fibrosis is a common pathological process resulted from various chronic hepatic injuries, which is characterized by an increase of extracelluarmatrix (ECM) deposition in the Disse’s space and the imbalance between synthesis and degradation of ECM [2]. The liver fibrosis is induced by viral hepatitis. In Friedman’s research, alcohol consumption, autoimmune disorders, drug-induced, helminthic infection, iron or copper overload and biliary obstruction may be also a major cause of morbidity and mortality of liver fibrosis worldwide [3]. And Giuseppe Poli suggested that reactive oxidant species likely contribute to both onset and progression of liver fibrosis [4]. As mentioned front, we can know that the pathogenesis of liver fibrosis is complex and diversity. But most of them involve the production of free radicals and oxidative stress [5]–[8]. CCl_4_ is a potent hepatoxin producing centrilobular hepatic necrosis which is widely used for animal models of liver fibrosis. It has been reported that CYP450 in rat liver activates CCl_4_ to a trichloromethyl free radical (CCl3• and/or CCl3OO•), and stimulates Kupffer cells to produce ROS, such as •O_2_^−^, H_2_O_2_ and •OH, which damage the liver [9]–[11]. These ROS are eliminated by antioxidant enzymes such as superoxide dismutase (SOD), glutathione peroxidase (GSH-Px) and CAT. Upregulation of many antioxidant enzymes in liver is mediated by Nrf2, which plays a pivotal role in the activation of ARE-driven antioxidant gene expression [12], [13]. Normally, Nrf2 is a member of the “‘cap’ ‘n’ collar” family of basic leucine zipper (bZIP) transcription factors, which is sequestered by its suppressor Kelch-like ECH-associated protein 1 (Keap1) in the cytoplasm when in an inactive state, but in an active state, translocated into the nucleus where it binds to the ARE and led to expression of target genes, upon cell stimulation [14]–[16]. In the research of Jiang T indicated that Nrf2 acts as a protective role against arsenic induced liver toxicity in six weeks *in vivo*. It showed that Nrf2 protects liver against arsenic by controlling a cellular antioxidant response through transcriptional upregulation of an array of down-stream genes, such as GCL, HO-1, GST, MRPs and NQO1 [17]. And Xu WH suggested that activation of Nrf2 may be also a novel strategy to prevent or ameliorate toxin-induced liver injury and liver fibrosis, demonstrated that Nrf2 can protect liver from ROS damage by regulating the Nrf2 target genes in hepatocytes, which encode enzymes involved in the detoxification of CCl_4_ and its metabolite, such as GST, NQO1 and GCLC [18]. But the research of Wang YP suggested that the protective role of Nrf2 against liver damage may not be related during a long-term of CCl_4_ administration in 8 weeks [13]. Therefore, Nrf2 is regarded as a protective role and known to act as a mediator in CCl_4_-induced liver fibrosis by regulated the antioxidant enzymes activity and relative genes expression, but its protective role is perhaps relative to the damage time. GA is one of the derivative products of Glycyrrhizic acid (GL). It is the most effective medicine in clinical, which is extracted from Glycirrhizaglabra. There are a lot of researches showed that GA has potent antiviral, antimutagenic, anti-inflammatory, anti-injury and antioxidant properties as well as liver protection [19]–[25]. It has been clinically used in the treatment of liver diseases. However, the mechanisms underlying the liver fibrosis elicited by GA in association with Nrf2 up-regulation remain poorly understood [26].

Hence, the aimed of this research was to study that liver fibrosis caused by oxidative stress induction by CCl_4_, and the involvement of Nrf2 activation in the reaction to this stress. Especially, the research on the relationship among Nrf2, its target genes (SOD3mRNA, CATmRNA, GPX mRNA) and the treatment of GA on liver fibrosis induced by CCl_4_ was completely blank.

Given these findings, we present a detail overview of the oxidative stress induction with activation of Nrf2 by CCl_4_, and attempt to elucidate the possible mechanism of GA on treatment of liver fibrosis induced by CCl_4_ that the up-regulation of Nrf2 associative with the enhancing expression of its target genes.

## Materials and Methods

### Chemicals and Reagents

The power of GA (HPLC >98%) was purchased from the local Chinese Natural additive Co., LTD-XIAN CHongXin. Silymarin was purchased from Madaus AG. CCl_4_ and peanut oil were purchased from MAGBIO Company in China. All other reagents were of analytic grade.

### Animals and Treatment

Five-week-old male Kunming mice (25–30 g, affiliated hospital animal center of ZhongShan University) were used in this experiment. The animals were allowed free access to Purina Rodent Chow and tap water ad libitum and randomly divided into six groups containing 8 animals per group. They were maintained in a controlled environment at 25±3°Cand 75±2% relatively humidity with a 12 h dark/light cycle, and acclimatized for one week before the experiment. All procedures were conducted in accordance with the Guidelines for the Care and Use of Laboratory Animals and approved by the Animal Care Committee of the Immunomodulation Research Center, GuangDong Medical College, Guangdong, and China. Mice in group I is control (Cont) and treatment with vehicle (i.g.) daily for 30 days. Mice from group II to group VI received CCl_4_ at dose of 6.4 g/kg respectively by subcutaneous injection under the skin between head and neck and then given treatment with either vehicle (group II, CCl_4_), silymarin 100 mg/kg/d (group III, Sil), or GA 25 mg/kg/d (group IV, GA25), GA 50 mg/kg/d (group V, GA50) and GA 100 mg/kg/d (group VI, GA100) for 30 days respectively. Before treatment, CCl_4_ dissolved in corn oil (12.8 g/kg BW, s.c.) was administrated under the skin between head and neck of mice to each group except Group I, and the following four dose of CCl_4_ was 6.4 g/kg BW in corn oil. But Group I (Cont.) was given corn oil with the same dosage with other groups every subcutaneous injection. The food was removed from the cage 12 h after the final treatment. Blood samples were collected from the eyeball vein plexus, and the livers were quickly excised from the mice. The blood samples were centrifuged to obtain serum at 3, 000×g for 15 min at 4°C. The excised livers were washed with cold phosphate buffer saline (PH 7.4), and pieces of the liver samples were fixed in 4% paraformaldehyde for histopathological examination. The remnants of the livers were stored at −80°C until the experimental use.

### Histopathological Examination

The fixed liver specimens were dehydrated in a graded alcohol series. Following xylene treatment, the specimens were embedded in paraffin blocks, cut into 5 µm thick sections, and placed on glass slides, then stained with hematoxyline-eosin (H-E), V-G (a collagen fiber dyeing) and Masson (a trichrome stain), according to standard procedure. To evaluate CCl_4_-induced histopathological changes, the stained tissue sample was examined under a light microscope of 40–200×. A minimum of 10 fields was scored per liver slice to obtain the mean value. The scoring system was devised in conjunction with the experienced liver histopathologies of Kerry Thompson to assess both qualitative and quantitative changes, and based on our previous experience with this model in our lab. Separate parameters were employed to score changes after administration with CCl_4_ and chronic liver fibrosis treatment with GA (Table 1 and 2). Specimens were scored blinded by the histologist and were also ranked blind for severity of fibrosis, using H-E, V-G and Masson sections [27]–[29].

**Table 1:** Scoring system for the treatment with GA on the CCl_4_-induced hepatotoxicity.

Histological Parameter	Score	Description of Lesion
Inflammatory infiltrate (HE)	0	absent
	1	Scanty cells with steatosis present at junction of necrotic zone
	2	Cells with hydropic and balloon degeneration regularly present
	3	Predominantly neutrophil clusters
	4	Predominantly mononuclear cell clusters

**Table 2:** Scoring system for the treatment with GA on the CCl_4_-induced liver fibrosis.

Histological Parameter	Score	Description of Lesion
Fibrosis (V-G/Masson)	0	absent
	1	Trace, slender septa present
	2	Mild, slender septa linking hepatic veins
	3	Moderate, Broad/well-developed septa
	4	Severe. cirrhosis

### Serum Analysis

The serum activity of MAO, GOT and GPT was measured to evaluate liver fibrosis. An autoanalyzer (Synergy2, Biotek, USA) was used in the experiments.

### Homogenate Preparation

Liver tissues were homogenized with exact buffer (m/v = 1∶9) according to the protocols of commercially available kits, and centrifuged at 3, 000×g for 15 min in a high-speed centrifuge (Eppendorf Cebtrifuge5415R, Eppendorf, Germany) at 4°C. The supernatants were collected to determine the activity of SOD, CAT, GSH-Px and MAO as well as the content of MDA.

### Lipid Peroxidation

The content of MDA, a compound produced during lipid peroxidation, was determined by the commercially available colorimetric assay kit (NanJingJianCheng Bioengineering Institute, Nanjing, China). Measurement of MDA was used as an indicator of lipid peroxidation [30]. This assay is based on the reaction of MDA with thiobarbituric acid (TBA). Two molecule of chromogenic reagent (2-Thiobarbituric acid) with one molecule of MDA to yield a stable chromophore at 95°C, forms a MDA-TBA_2_ adduct that absorbs strongly at 532 nm.

100 µl aliquot of sample solution and MDA standard solution were added to microcentrifuge tube respectively, and added the reagents on the kits into each tube and vortexed, each microcentrifuge tube was incubated at 95°C for 40 min and centrifuged at 4,000×g for 10 min. Then 300 µl of the supernatant was transferred to a microplate and the absorbance measured at 532 nm using a multi-detection microplate reader (Synergy2, Biotek, USA). The level of MDA is expressed as nM MDA/mg homogenate protein.

### Antioxidant Enzymes Activity

SOD, GSH-Px and CAT activities were measured according to the protocols of commercially available kits (JianCheng Bioengineering Institute, Nanjing, China). SOD activity was evaluated by utilizing a tetrazolium salt for detection of superoxide radicals, generated by xanthine oxidase and hypoxanthine. One unit of SOD is defined as the amount of enzyme needed to exhibit 50% dismutation of the superoxide radical at 37°C. The reaction product was measured at 450 nm using a Synergy2 Automatic microplate reader. SOD activity in the liver tissues was expressed as units per milligram protein (U/mgprot). GSH-Px activity was based on the reaction of GSH transforms into GSSG. Reduced glutathione in liver homogenate was determined by reaction with 1, 2-dithio-bis nitro benzoic acid (DTNB). Briefly, 1 mol DTNB with 2 mol GSH reacted together to 1 mol 5, 5′-dithiobis (2-nitrobenzoic acid) with an intense yellow color, measured spectrophotometrically at 412 nm using a Synergy2 Automatic microplate reader. Results were expressed as µmol GSH/mg protein. CAT activity was based on the reduction of the H_2_O_2_. 0.1 ml 1% homogenate was incubated in 1.0 ml substrate (65 µmol per ml hydrogen peroxide in 60 mmol/L sodium-potassium-phosphate buffer, PH 7.4) at 37°C for 60 s. One unit catalase decomposes 1 µmol of hydrogen peroxide/min under this condiction. Then the enzymatic reaction was stopped with 1.0 ml of 32.4 mmol/L ammonium molybdate ((NH_4_)_6_Mo_7_O_24_•4H_2_O). And the yellow complex of molybdate and hydrogen peroxide was measured spectrophotometrically at 405 nm. During the test, the control tube and sample tube should do at the same time.

### MAO Activity

MAO is a family of enzymes that catalyze the oxidation of monoamines [31], [32]. They are found bound to the outer membrane of mitochondria in most cells of the body. The enzyme was originally discovered by Mary Bernheim in the liver and was named tyramine oxidase [33], [34]. They belong to the protein family of flavin-containing amine oxidoreductases. And the MAO in the serum is hydrosoluble. It is similar to connective tissue’s, but different to mitochondria’s. The MAO activity in serum is positively correlated to that in homogenate. Both of them are also positively correlated to liver fibrosis. The MAO activity is measured spectrophotometrically at 242 nm using a Synergy2 Automatic microplate reader, according to the protocols of commercially available kits (JianCheng Bioengineering Institute, Nanjing, China).

### Preparation of Nuclear Fractions from Liver

Microsomes were prepared as the guidance of Nucleoprotein protein Extraction kits (Sangon Biotech Co., LTD, Shanghai, China). The liver was washed in phosphate buffered saline twice, and then homogenized in three volumes (w/v) of the Hypotonic Buffer containing 5 µl phosphatase inhibitor, 10 µl PMSF and 1 µl DTT, ultrasound broken four times, 30 s per time. Then ice-bathed for 10 min, the liver homogenate was centrifuged at 3,000×g for 5 min in a high-speed centrifuge (Eppendorf Cebtrifuge5415R, Germany) at 4°C. After centrifuged in two volumes (w/v) of the same Hypotonic Buffer, the sediment was centrifuged at 5,000×g for 5 min. Then, the sediment washed and resuspended in the same volume (w/v) of the Lysis Buffer containing 5 µl phosphatase inhibitor, 10 µl PMSF and 1 µl DTT. After ice-bathed for 20 min, the suspension was centrifuged at 15,000× g for 10 min. The supernatant was defined as a nucleoprotein and stored at −80°C until used. Protein concentration was measured by BSA^Boster^ protein assay kit.

### Preparation of Cytoplasm Fractions from Liver

Microsomes were prepared according to the protocol of Cytoplasmic and membranal protein Extraction kits (Sangon Biotech Co., LTD, Shanghai, China). The liver was washed in phosphate buffered saline twice and centrifuged at 700×g for 3 min in a high-speed centrifuge at 4°C. Then, the sediment was homogenized in ten volumes (w/v) of the Hypotonic Buffer containing 5 µl phosphatase inhibitor, 10 µl PMSF, 1 µl DTT and 1 µl protease inhibitor, ultrasound broken six times, 20 s per time. Then ice-bathed for 10 min, the liver homogenate was centrifuged at 3,000 rpm for 10 min in a high-speed centrifuge at 4°C. Then the supernatant was transferred to another tube and centrifuged at 13,000 rpm for 75 min. The supernatant was defined as a cytoplasmic protein and stored at −80°C until used. Protein concentration was measured by BSA^Boster^ protein assay kit.

### Western Blot Analysis for Nrf2

Western blotting was performed by using the standard method. Equal amounts of proteins were separated by 10% SDS-poyacrylamide gel electrophoresis and electro-transferred to an Immun-Blot^solarbio^ PVDF membrane (0.22 µm pore size, Solarbio). Membranes were blocked overnight at 4°C in Phosphate Buffered Saline (PBS) with 5% w/v skimmed milk powder (Sangon, Shanghai, China), then the membranes were incubated at 4°C with primary antibody diluted in the normal saline contained 0.025% v/v Tween-20 (GAPDH 1∶2000, Nrf2 1:200, Santa Cruz Biochemistry, INC. CA, USA) overnight. After washed with 0.025% v/v Tween-20 in PBS, the membrane was incubated with anti-rabbit IgG AP-linked secondary antibody for 2 h at room temperature and then washed with the same buffer. The blots in the samples were quantified by densitometry analysis using Quantity One software. All data for three independent experiments were expressed as relative intensity compared to the control group for statistical analysis.

### QRT-PCR Analysis

Total RNA from liver tissues were extracted using Trizol (Invitrogen, USA). The concentration of total RNA in each sample was quantified spectrophotometrically at 260 nm. The integrity of each RNA sample was evaluated by formaldehyde-agarose gel electrophoresis before analysis. Then equal amounts of RNA (1 µg) were reverse-transcribed into cDNA using the Transcriptor First Strand cDNA synthesis Kit (Promega, USA) according to the manufacturer’s instructions, and the resulting cDNA was used for real-time PCR analysis using SYBR® Green PCR Master Mix in a ABI7500 Fast Real-Time PCR System (USA).The RT-PCR was performed under the instruction of RT-PCR kit (DRR820A, TAKARA) and all reactions were performed in triplicate, and PCR runs were repeated twice. Data were analyzed using ABI7500 biosystems. The data presented were relative mRNA levels normalized to GAPDH, and the value from the control group was set as 1. The target mRNA was performed with specific primers provided by TAKARA. Primer sequences described as followed: SOD3, 5′CTTGTTCTACGGCTTGCTACTG3′ and 5′ ATGCGTGTCGCCTATCTTCT3′; CAT, 5′ CCAGTGCGCTGTAGATGTGAAAC3′and 5′ GGTGGACGTCAGTGAAATTCTTG3′; GPX2, 5′CCACTGTTTCCCCTGAGCA3′ and 5′CAGACTTAGAGCCCCAAGCA3′; GAPDH, 5′ GGTGAAGGTCGGTGTGAACG3′ and 5′CTCGCTCCTGGAAGATGGTG3′.

### FeCl2-ascorbic Acid Stimulated Lipid Peroxidation Assay

A murine liver homogenate of the young male mice was used. The reaction mixture was composed of 0.25 ml of the liver homogenate, 0.05 ml of 0.1 mM ascorbic acid, 0.1 ml of Tris-HCl buffer (PH 7.2), 0.05 ml of 4 mM FeCl_2_ and 0.05 ml of a solution with various concentrations of GA (dissolved in dimethyl-sulfoxide). Control was treated only with dimethylsulfoxide, the final concentration of which never exceeded 0.1%. And this concentration did not have any noticeable effect on the assay systems [35]. After incubated in 37°C for 120 min, the lipid peroxidation products were measured by MDA assay kit (JianCheng Bioengineering Institute, Nanjing, China). 1, 1, 3, 3-Tetraethoxypropan was used as a standard for the calibration of MDA.

### Assay of DPPH Radical Scavenging Activity

The antioxidant and free radical scavenging activity of GA was determined by the stable free radical diphenylpicrylhydrazyl (DPPH), in terms of the mechanism of its treatment of liver fibrosis. The reaction was composed of 3.9 ml of 25 mg/L DPPH and 0.1 ml of a solution with various concentrations of GA (all dissolved in ethanol). Control was treated only with ethanol. The products were measured at 517 nm after 5 min reaction. Ascorbic acid was used as a reference inhibitor. All the datas for three independent experiments were expressed by a DPPH curve [36], [37].

### Statistical Analysis

Data bars represent the means±SD (standard deviations) for at least three independent experiments in all cases. Two group comparisons were evaluated by Student’s *t*-test as appropriate. Differences were considered statistically significant when the *p* value was <0.05.

## Results

### Effect of GA on CCl4-induced Hepatotoxity

**Figure 1:**
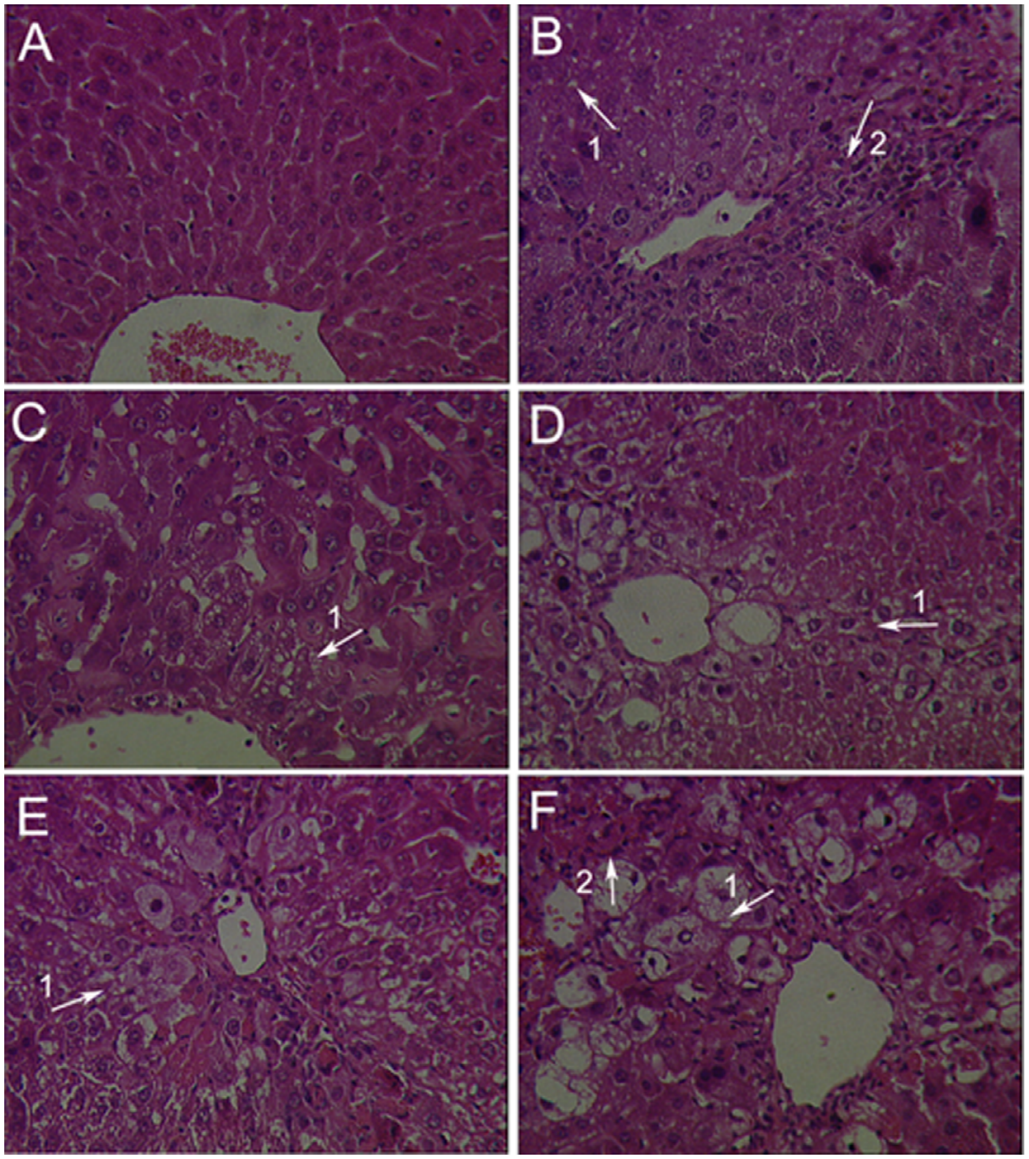
Effects of GA on histopathological changes by CCl_4_ in mice were evaluated in sections stained with hematoxyline-eosin. Mice in all groups were treated as the same with the front method. The animals were sacrificed 24 h after the last CCl_4_ administration and the liver was removed, fixed and embedded in paraffin. Sections were stained with hematoxyline-eosin (H-E, 200×). (A) Liver tissue of a control mouse. (B) Liver tissue of a mouse treated with CCl_4_, presenting severe hepatocyte necrosis with neutrophil clusters and mononuclear cells infiltration (arrow 2) around the portal vein. (C) Liver tissue of a mouse treated with silymarin (100 mg/kg, i.g.), showing mild hepatocyte necrosis with inflammatory cell infiltration and steatosis (arrow 1) around the portal vein. (D) Liver tissue of a mouse treated with GA (25 mg/kg, i.g.), showing moderate hepatocyte necrosis with inflammatory cell infiltration and moderate steatosis (arrow 1). (E) Liver tissue of a mouse treated with GA (50 mg/kg, i.g.), showing mild steatosis (arrow 1) around centrilobular and midzone region. (F) Liver tissue of a mouse treated with GA (100 mg/kg, i.g.), showing mild hepatocyte necrosis with inflammatory cell infiltration (arrow 2) and severe steatosis (arrow 1).

**Figure 2:**
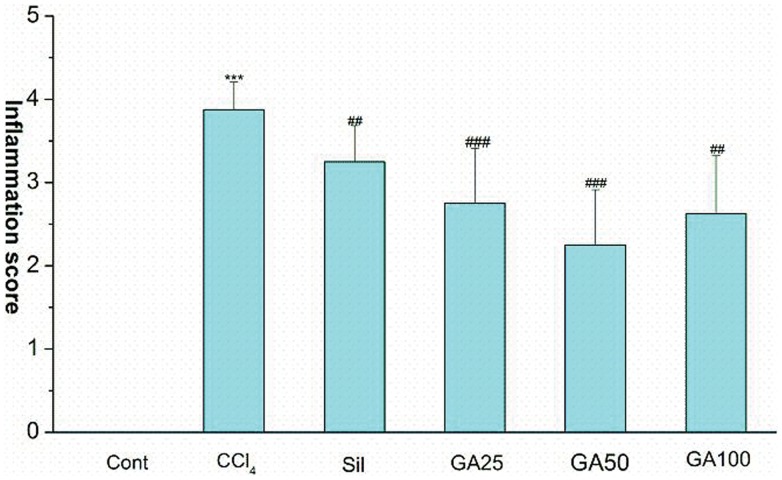
The inflammation score was evaluated in the livers of surviving animals by certified pathologist in a blinded fashion. Cont, normal control; CCl_4_, CCl_4_ alone; Sil, CCl_4_+100 mg/kg silymarin; GA25, CCl_4_+25 mg/kg GA; GA50, CCl_4_+50 mg/kg GA; GA100, CCl_4_+100 mg/kg GA. Datas are presented as the mean ± SD (n = 8) in each group. *** Significantly different from the control at *p*<0.001; ^##^ Significantly different from the CCl_4_ at *p*<0.01; ^###^ Significantly different from the CCl_4_at *p*<0.001.

Serum GOT, GPT and histopathological were examined to evaluate the effect of GA on CCl_4_- induced hepatotoxicity in mice. As showed in Table 3, the group administrated with CCl_4_ only (model group) caused hepatotoxicity in mice indicated by marked increased GOT and GPT serum levels. GA and silymarin reversed the CCl_4_-induced elevation of serum GOT and GPT levels to some extent. Histopathological studies showed that, compared to the control group, CCl_4_ induced severe degeneration in the hepatocytes with extensive inflammatory cell infiltration around the central vein, portal area as well as focal necrosis (Fig. 1B). According to microscopic examinations, severe hepatic lesions induced by CCl_4_ were remitted by the administration of GA to some extent, which were in good agreement with the results of the serum aminotransferase activities, GA can reverse the increasing level of GOT/GPT in serum induced by CCl_4_. In GA 25 mg/kg treated group (Fig. 1D), showing moderate hydropic degeneration of hepatocytes. And hepatocyte necrosis was merely diminished, and disappeared nearly in the GA 50 mg/kg treated group (Fig. 1E), showing a significantly reduction of the level of hydropic degeneration and necrosis. But in GA 100 mg/kg group (Fig. 1F), hepatocyte necrosis was severe and showing ballooning degeneration. In Fig. 2, it was showed that GA (25 and 50 mg/kg) significantly decreased inflammation score. These results showed that GA treated CCl_4_-induced hepatotoxicity to some extent.

**Table 3:** Effects of the treatment with GA on the CCl_4_-induced liver fibrosis and lipid peroxidation.

Groups	Serum GOT(U/L)	Serum GPT(U/L)	Lipid peroxidation(MDA: nM/mgpro)
**Cont**	8.58±2.70^b^	7.63±0.79^b^	2.42±0.53^b^
CCl_4_	94.50±11.49^a^	49.03±6.47^a^	3.30±0.45^a^
Sil	15.22±3.11a, b	29.90±4.03a, b	2.96±0.59
GA25	15.81±3.42a, b	37.40±10.09a, b	3.00±0.16^a^
GA50	18.36±6.10a, b	29.21±4.40a, b	2.84±0.56^b^
GA100	23.11±4.08a, b	40.05±11.06a, b	3.11±0.56^a^

The mice were treated with GA, silymarin or vehicle every day for 4 weeks on the other day of the first double-dosage administration with CCl_4._ The CCl_4_ dissolved in corn oil (6.4 g/kg of body weight, s.c.) was administrated under the skin between head and neck to each group every 6 days during this month, except Cont. The animals were sacrificed 24 h after the last CCl_4_ administration. Hepatotoxicity was determined by quantifying the serum activities of GPT and GOT as well as hepatic lipid peroxidation. Cont, normal control; CCl_4_, CCl_4_ alone; Sil, CCl_4_+100 mg/kg silymarin; GA25, CCl_4_+25 mg/kg GA; GA50, CCl_4_+50 mg/kg GA; GA100, CCl_4_+100 mg/kg GA. Data are presented as the mean±SD (n = 8) in each group.

a Significantly different from the control at *p*<0.05.

b Significantly different from the CCl _4_ at *p*<0.05.

### Effect of GA on CCl4-induced Liver Fibrosis

**Figure 3:**
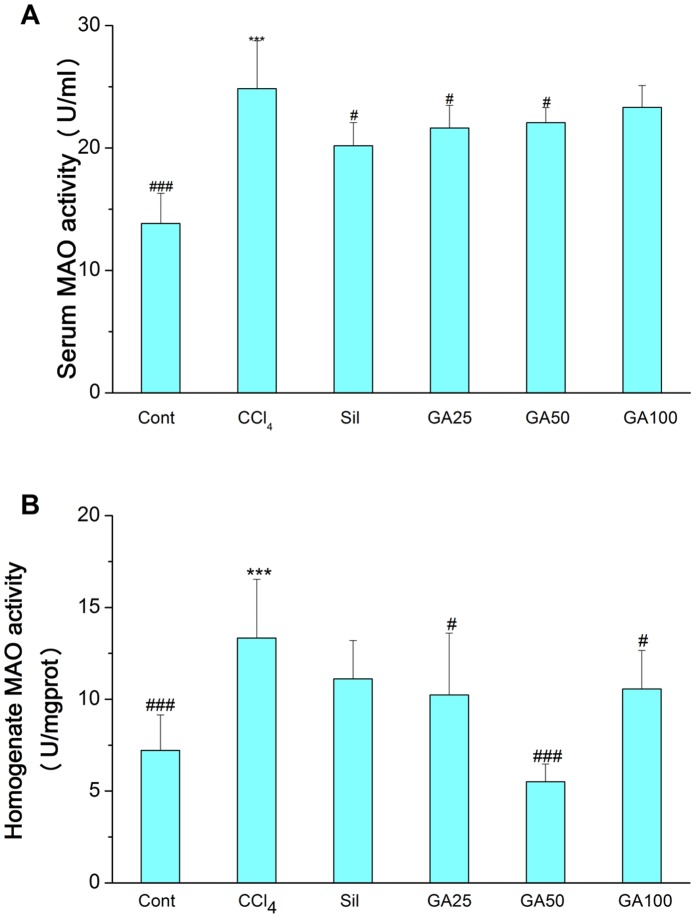
Effects of GA on serum and homogenate MAO changes by CCl_4_ in mice. Mice in all groups were treated as the same with the front method. Liver fibrosis was determined by quantifying the serum activities of MAO (Fig. 3A) as well as liver homogenate MAO (Fig. 3B). Cont, normal control; CCl_4_, CCl_4_ alone; Sil, CCl_4_+100 mg/kg silymarin; GA25, CCl_4_+25 mg/kg GA; GA50, CCl_4_+50 mg/kg GA; GA100, CCl_4_+100 mg/kg GA. Datas are presented as the mean± SD (n = 8) in each group. *** Significantly different from the control at *p*<0.001; ^#^ Significantly different from the CCl_4_ at *p*<0.05; ^###^ Significantly different from the CCl_4_ at *p*<0.001.

**Figure 4:**
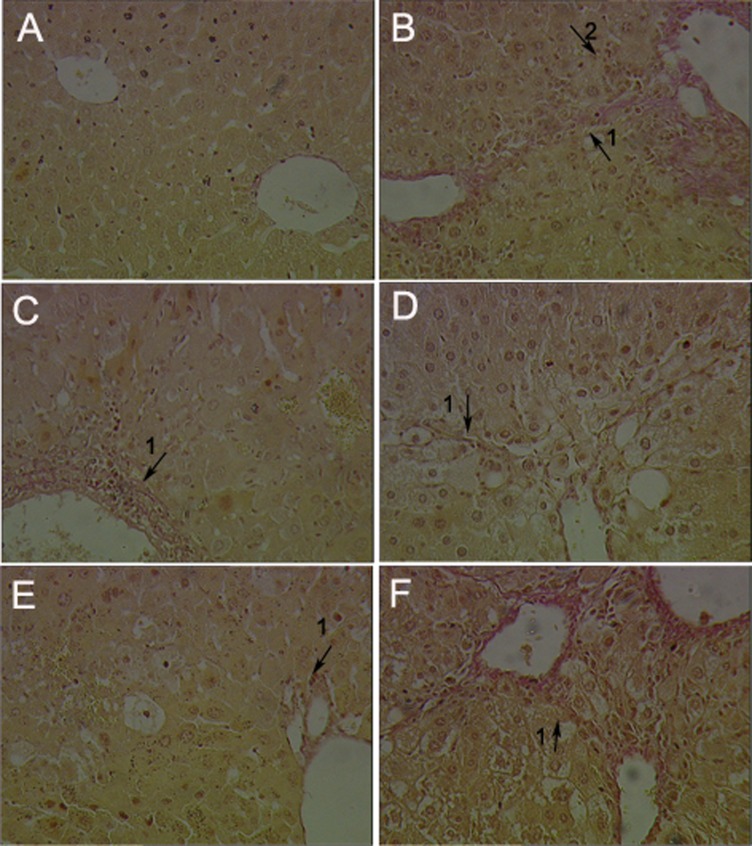
Effects of GA on histopathological changes by CCl_4_ in mice were evaluated in sections stained with collagenous fiber. Mice in all groups were treated as the same with the front method. The animals were sacrificed 24 h after the last CCl_4_ administration and the liver was removed, fixed and embedded in paraffin. Sections were stained with collagenous fiber (V-G, 200×). (A) Liver tissue of a control mouse. (B) Liver tissue of a mouse treated with CCl_4_, numerous fibrocytes appeared at the periphery of the lesions, and the collagen fibers became longer and thicker, presenting severe liver fibrosis and severe hepatocyte necrosis with inflammatory cell infiltration around the portal vein. (C) Liver tissue of a mouse treated with silymarin (100 mg/kg, i.g.), showing broad-develop septa and moderate liver fibrosis around the portal vein. (D) Liver tissue of a mouse treated with GA (25 mg/kg, i.g.), showing slender septa linking hepatic vein and mild hepatic fibrogenesis. (E) Liver tissue of a mouse treated with GA (50 mg/kg, i.g.), showing mild fibrosis around centrilobular and midzone region. (F) Liver tissue of a mouse treated with GA (100 mg/kg, i.g.), showing moderate fibrosis. Arrow 1 shows collagen fibers which was stained red, while arrow 2 shows inflammatory cell infiltration.

**Figure 5:**
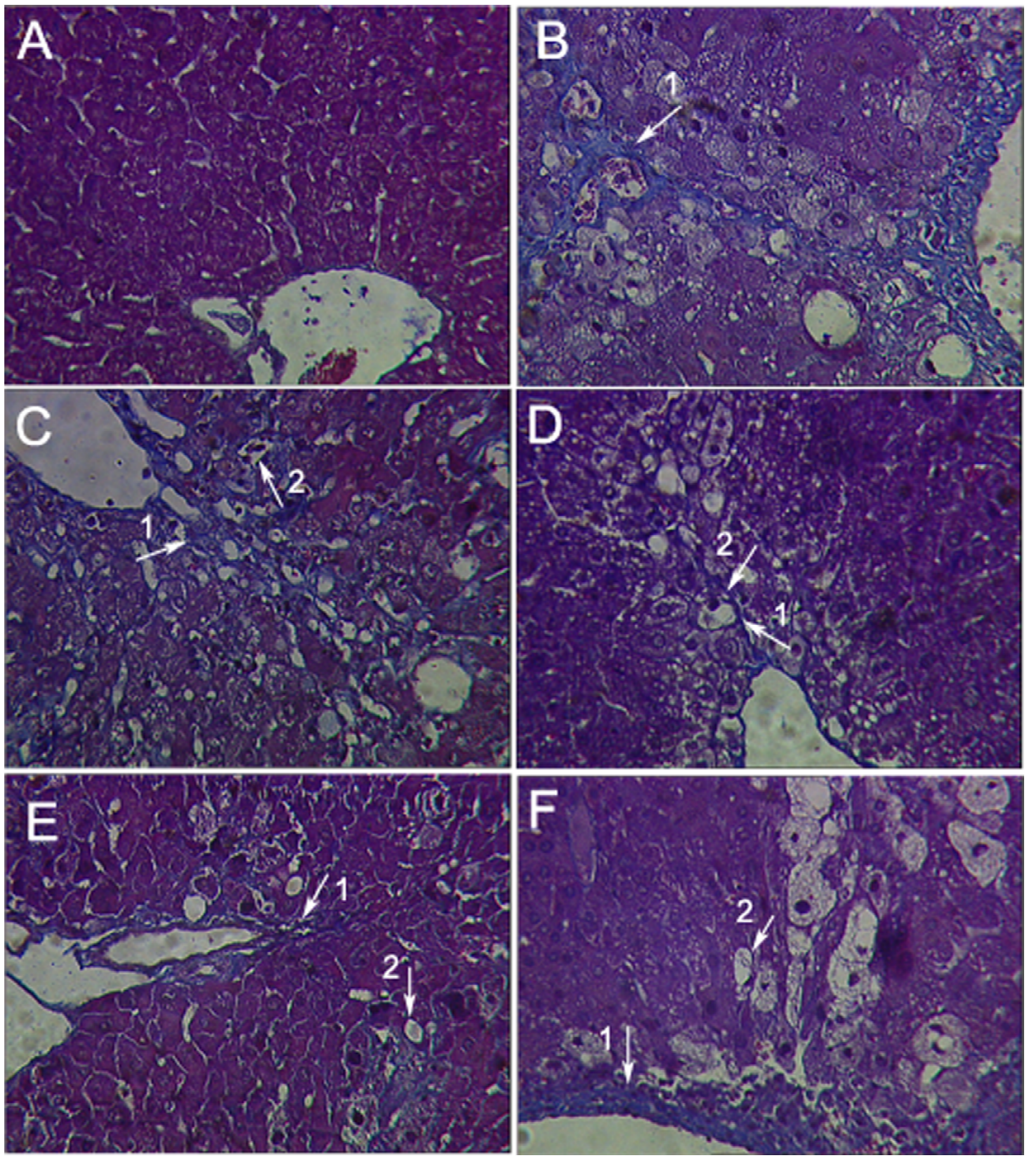
Effects of GA on histopathological changes by CCl_4_ in mice were evaluated in sections stained with Masson. Mice in all groups were treated as the same with the front method. The animals were sacrificed 24 h after the last CCl_4_ administration and the liver was removed, fixed and embedded in paraffin. Sections were stained with Masson (200×). (A) Liver tissue of a control mouse. (B) Liver tissue of a mouse treated with CCl_4_, presenting severe liver fibrosis (arrow 1) and ballooning degeneration (arrow 2) aroud the portal vein. (C) Liver tissue of a mouse treated with silymarin (100 mg/kg, i.g.), presenting well-developed septa, showing moderate liver fibrosis (arrow 1) and hepatocyte necrosis with inflammatory cell (arrow 2) infiltration around the portal vein. (D) Liver tissue of a mouse treated with GA (25 mg/kg, i.g.), presenting slender septa linking hepatic veins, showing mild liver fibrosis (arrow 1) and severe sreatosis (arrow 2). (E) Liver tissue of a mouse treated with GA (50 mg/kg, i.g.), presenting slender septa, showing mild fibrosis (arrow 1) and sreatosis (arrow 2) around centrilobular and midzone region. (F) Liver tissue of a mouse treated with GA (100 mg/kg, i.g.), showing severe fibrosis (arrow 1) and sreatosis (arrow 2). Arrows show collagen fibers, which were stained blue (Masson trichrome staining).

**Figure 6:**
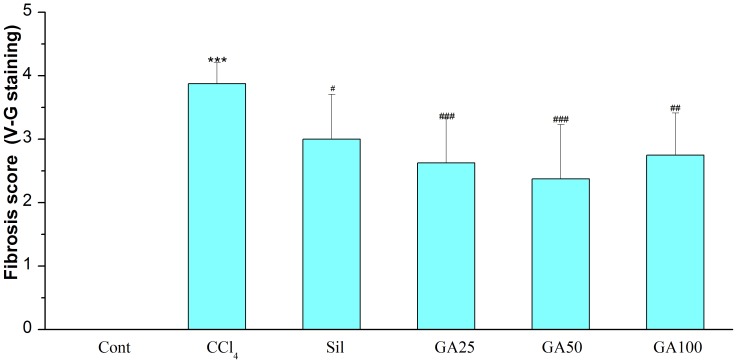
Fibrosis score. Fibrosis score was evaluated in the liver sections stained with V-G of surviving animals by certified pathologist in a blinded fashion. Cont, normal control; CCl_4_, CCl_4_ alone; Sil, CCl_4_+100 mg/kg silymarin; GA25, CCl_4_+25 mg/kg GA; GA50, CCl_4_+50 mg/kg GA; GA100, CCl_4_+100 mg/kg GA. Datas are presented as the mean ± SD (n = 8) in each group. *** Significantly different from the control at *p*<0.001; ^#^ Significantly different from the CCl _4_ at *p*<0.05; ^##^ Significantly different from the CCl_4_ at *p*<0.01; ^###^ Significantly different from the CCl_4_ at *p*<0.001.

**Figure 7:**
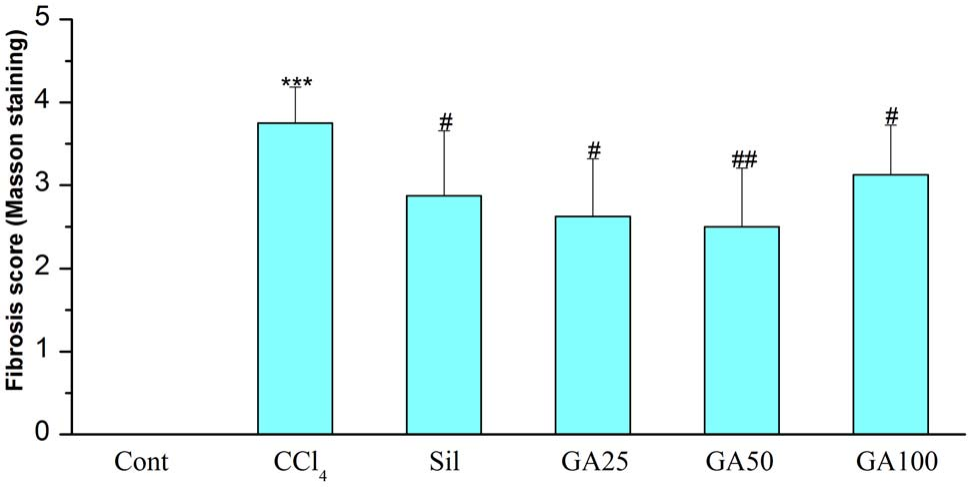
Fibrosis score. Fibrosis score was evaluated in the liver sections stained with Masson of surviving animals by certified pathologist in a blinded fashion. Cont, normal control; CCl_4_, CCl_4_ alone; Sil, CCl_4_+100 mg/kg silymarin; GA25, CCl_4_+25 mg/kg GA; GA50, CCl_4_+50 mg/kg GA; GA100, CCl_4_+100 mg/kg GA. Datas are presented as the mean ± SD (n = 8) in each group. *** Significantly different from the control at *p*<0.001; ^#^ Significantly different from the CCl_4_ at *p*<0.05; ^##^ Significantly different from the CCl_4_ at *p*<0.01.

Serum MAO, homogenate MAO and histopathological were examined to evaluate the effect of GA on CCl_4_-induced liver fibrosis in mice. As shown in Fig. 3, CCl_4_ caused liver fibrosis by marked increased serum MAO and homogenate MAO. GA and silymarin treatment relieved the CCl_4_-induced elevation of serum and homogenate MAO levels. Histopathological studies showed that CCl_4_ compared to the control, induced severe degeneration in the hepatocytes with fibrosis around the central vein as well as focal necrosis (Fig. 4B; Fig. 5B). According to microscopic examinations, severe liver fibrosis induced by CCl_4_ was reduced by the administration of GA to some extent, which was in good agreement with the results of the serum MAO and homogenate MAO. In GA 25 and 100 mg/kg group (Fig. 4D; Fig. 5D and Fig. 4F; Fig. 5F), liver fibrosis was mildly diminished, and merely disappeared in the GA 50 mg/kg (Fig. 4E; Fig. 5E), showing a significantly suppression of hepatic fibrogenesis but severe to moderate hydropic degeneration of hepatocytes. These results were consistent with the fibrosis scores showed in Figure 6 and Figure 7. GA (25, 50 and 100 mg/kg) decreased fibrosis scores to different extent.

### Effect of GA on CCl4-induced Hepatic Lipid Peroxidation

To evaluate the effect of GA treatment on CCl_4_-induced liver lipid peroxidation, the levels of MDA, an index of oxidative damage and one of the decomposition products of peroxidased polyunsaturated fatty acids, were monitored. As showed in Table 3, a significant increase of MDA in the CCl_4_-treated group confirmed that oxidative damage had been induced. Consistent with the serum levels of GPT and GOT, treatment with GA (25, 50 mg/kg) and silymarin were significantly decreased CCl_4_-induced lipid peroxidation (Table 3). In the mice of group IV to VI treated with GA at doses of 25, 50 and 100 mg/kg in the course of the CCl_4_ challenge was observed to dose-dependently reverse the CCl_4_-induced alteration of MDA by 9.1%, 13.9%, 5.8%.

### Effect of GA on CCl4-induced Changes in the Levels of Hepatic Antioxidant Enzyme Activities

It has been suggested that SOD, GSH-Px and CAT served as the detoxifying system for the prevention of damage caused by ROS, and play pivotal roles in the scavenging of free radicals. To provide insight into the relationship between the antifibrosis effect and the antioxidant effect of GA, the activities of these antioxidant enzymes were analyzed. As shown in Table 4, CCl_4_ induced substantial modifications to the hepatic antioxidative enzymes and the decreased hepatic SOD, GSH-Px and CAT activities as the result of CCl_4_ subcutaneous injection were significantly elevated in the GA and silymarin groups, and the effect was strengthened with GA in the concentration of 25 and 50 mg/kg. Interestingly, the CAT activities of the 25 mg/kg and 50 mg/kg GA treated groups were higher than those of the CCl_4_-treated group, being at even higher than those of the control group. The SOD activities of the 25 mg/kg and 50 mg/kg GA group were much higher than those of the CCl_4_-treated group. What’s more, the GSH-Px activities of GA groups were higher than those of the CCl_4_-treated group to some extent. All were shown in Table 4. Taken together, these results indicate that treatment with GA attenuated the changes in antioxidant enzyme activities induced by the administration of CCl_4_.

**Table 4:** Antioxidative effects of the treatment with GA on the CCl_4_-induced liver fibrosis.

Groups	SOD(U/mgprot)	CAT(U/mgprot)	GSH-Px(µmol/mgprot)
Cont	76.64±21.16^b^	51.34±4.01^b^	131.70±16.51^b^
CCl_4_	48.15±20.30^a^	44.50±8.46^a^	77.26±9.49^a^
Sil	127.62±17.15^b^	44.54±7.12	85.14±13.98^b^
GA25	111.09±24.27^b^	69.16±17.71^b^	95.59±10.40^b^
GA50	70.91±21.31^b^	71.68±7.34^b^	85.18±7.08^b^
GA100	40.80±14.56	52.55±8.92	85.81±10.07^b^

The mice were treated with GA, silymarin or vehicle every day for 4 weeks on the other day of the first double-dosage administration with CCl_4._ Then every 6 days during this month, CCl_4_ dissolved in corn oil (6.4 g/kg of body weight, s.c.) was administrated under the skin between head and neck to each group, except Cont. The activities of antioxidant enzyme in the liver were determined. Cont, normal control; CCl_4_, CCl_4_ alone; Sil, CCl_4_+100 mg/kg silymarin; GA25, CCl_4_+25 mg/kg GA; GA50, CCl_4_+50 mg/kg GA; GA100, CCl_4_+100 mg/kg GA. Data are presented as the mean± SD (n = 8) in each group.

a Significantly different from the control at *p*<0.05.

b Significantly different from the CCl_4_ at *p*<0.05.

### Effects of GA on Hepatic Nrf2 Content

**Figure 8:**
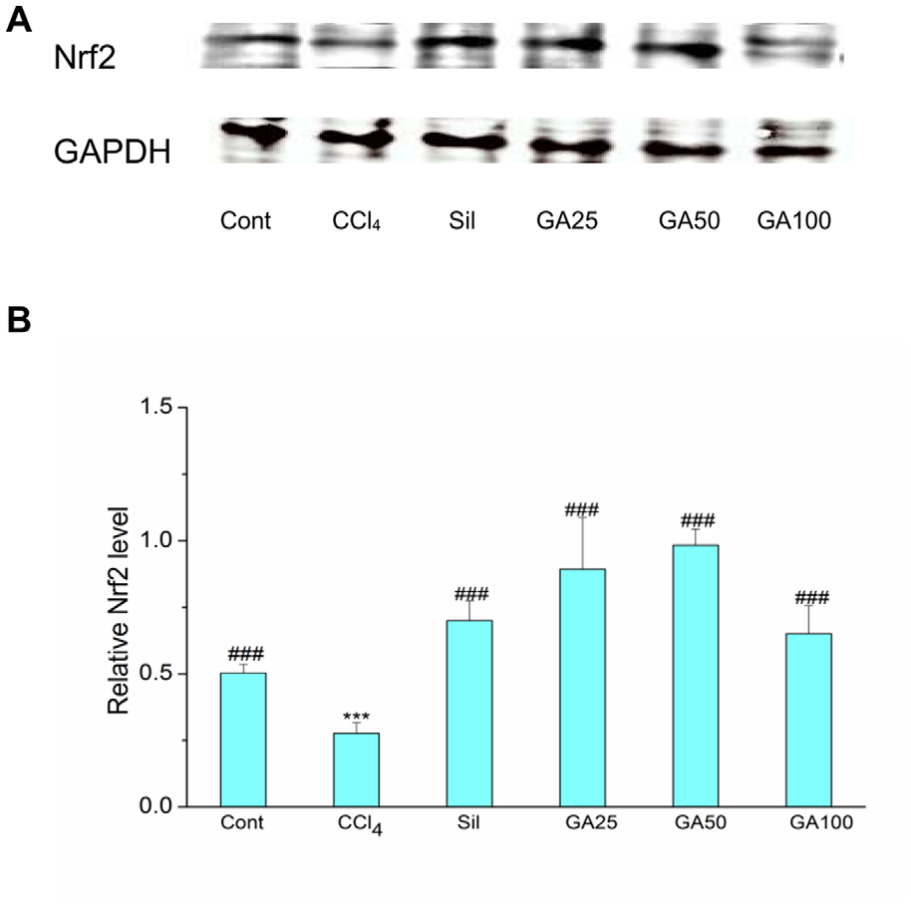
Effects of GA on CCl_4_ bioactivation related Nrf2 expression in cytoplasm. Mice in all groups were treated as the same with the front method. The animals were sacrificed 24 h after the last CCl_4_ administration. (A) The expression of Nrf2 and GAPDH in the liver microsomes was determined by western blotting. GAPDH was used as an internal control. (B) Quantitative analysis of the Nrf2 proteins. Cont, normal control; CCl_4_, CCl_4_ alone; Sil, CCl_4_+100 mg/kg silymarin; GA25, CCl_4_+25 mg/kg GA; GA50, CCl_4_+50 mg/kg GA; GA100, CCl_4_+100 mg/kg GA. Data are presented as the mean± SD for three independent experiments, performed in triplicate. *** Significantly different from the control at *p*<0.001; ^###^ Significantly different from CCl_4_ at *p*<0.001.

**Figure 9:**
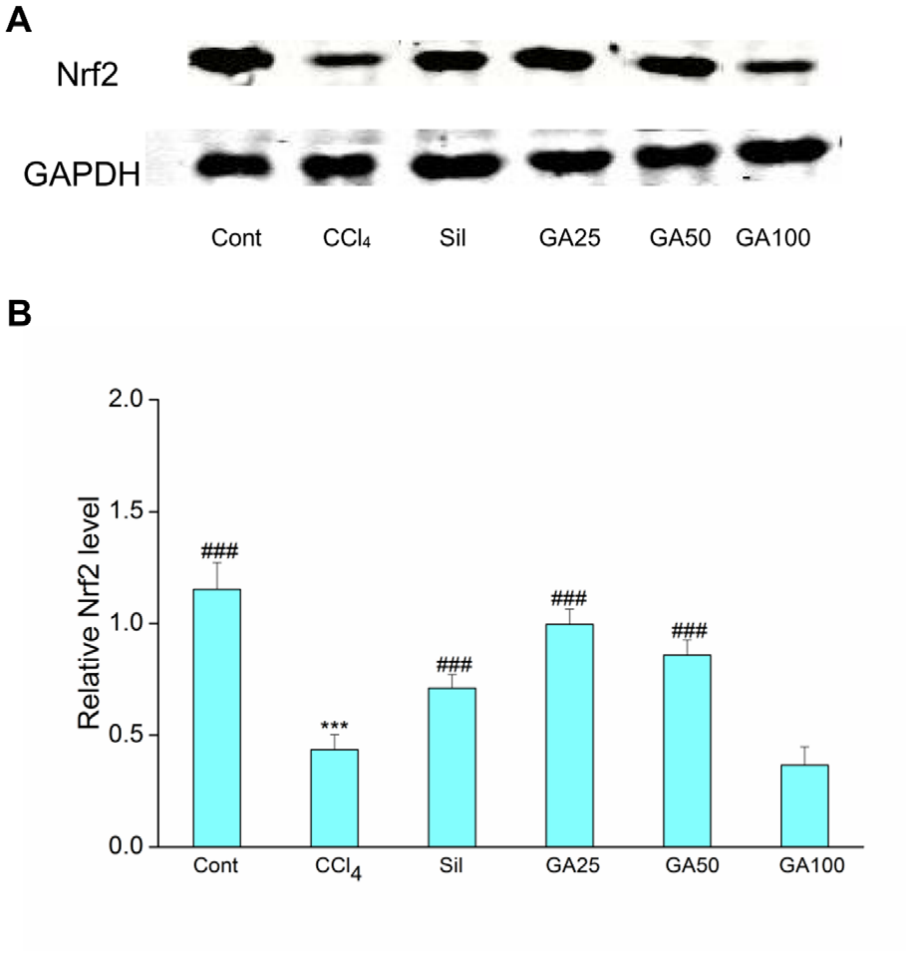
Effects of GA on CCl_4_ bioactivation related Nrf2 expression in nuclear. Mice in all groups were treated as the same with the front method. The animals were sacrificed 24 h after the last CCl_4_ administration. (A) The expression of Nrf2 and GAPDH in the liver microsomes was determined by western blotting. GAPDH was used as an internal control. (B) Quantitative analysis of the Nrf2 proteins. Cont, normal control; CCl_4_, CCl_4_ alone; Sil, CCl_4_+100 mg/kg silymarin; GA25, CCl_4_+25 mg/kg GA; GA50, CCl_4_+50 mg/kg GA; GA100, CCl_4_+100 mg/kg GA. Data are presented as the mean± SD for three independent experiments, performed in triplicate. *** Significantly different from the control at *p*<0.001; ^###^ Significantly different from CCl_4_ at *p*<0.001.

Nrf2 plays a key role in the activation of antioxidants enzymes by regulating their transcription [33]. Therefore, western blotting analysis was performed to examine the effect of GA on Nrf2 protein expression. As shown in Fig. 8, the Nrf2 of the mice receiving CCl_4_ alone showed a lower expression compared to the control mice in the cytoplasm. In contrast, the mice give administration with GA and silymarin showed a high level of Nrf2 with respect to that in the mice of control group. And the expression of Nrf2 in nucleoprotein was similar to it. As shown in Fig. 9, the mice receiving CCl_4_ alone showed a dramatic downregulation in the Nrf2 protein levels compared to the control mice in the nucleoprotein. Instead, the mice give administration with GA and silymarin markedly increased the levels of Nrf2 with respect to that in the mice intoxicated with CCl_4_ alone. The restoration of Nrf2 by GA supplementation implies that GA could have a hepatoprotective effect which led to recovery from CCl_4_-induced liver fibrosis.

### Effects of GA on Nrf_2_ Targeted mRNA Expression in Liver

**Figure 10:**
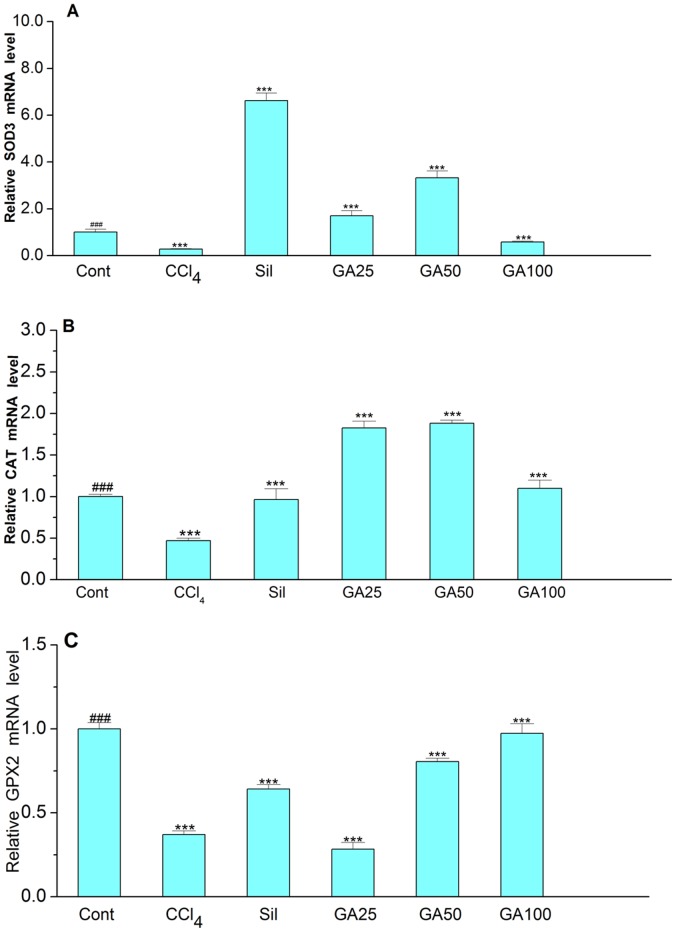
Effect of GA on the mRNA expression levels of SOD3, CAT, and GPX2 genes. Total RNA was extracted from mice liver tissues of GA-treated, CCl_4_-administrated, silymarin-treated, and control group animals after sacrificed. qRT-PCR was performed for analysis of mRNA expression levels of SOD3, CAT, and GPX2. (A) qRT-PCR results for analysis of SOD3 mRNA. (B) qRT-PCR results for analysis of CAT mRNA. (C) qRT-PCR results for analysis of GPX2 mRNA. Cont, normal control; CCl_4_, CCl_4_ alone; Sil, CCl_4_+100 mg/kg silymarin; GA25, CCl_4_+25 mg/kg GA; GA50, CCl_4_+50 mg/kg GA; GA100, CCl_4_+100 mg/kg GA. Data are presented as the mean± SD for three independent experiments, performed in triplicate.*** Significantly different from the control at *p*<0.001; ^###^ Significantly different from CCl_4_ at *p*<0.001.

To determine the mechanism of how GA protects against CCl_4_-induced liver fibrosis by Nrf2, CAT, GPX2 and SOD3 mRNA were quantified in livers of the six groups’ mice (Fig. 10). The SOD3 level of the six groups was shown in Fig. 10A. The SOD3 level was four-fold higher in control group, five -fold higher in silymarin group and 25 mg/kg GA group, ten-fold higher in 50 mg/kg GA group but twice higher in 100 mg/kg GA group, compared with the models. As shown in Fig. 10B, without CCl_4_ the basal level of CAT was 53% higher than the model group. Compared with the model group, the level of CAT was 76% higher in Silymarin, three -fold higher in the 25 mg/kg GA group and 84.3% higher in the 50 mg/kg GA group. But the level of CAT on the 100 mg/kg GA group was twice as much as the model group. Similarly, the level of GPX2 in control group was 62.9% higher than that in the model group. The level of GPX2 in Silymarin group was 72.9% higher than the model group. And that level in 50 mg/kg and 100 mg/kg GA group were twice higher than model group. But the level of GPX2 in 25 mg/kg GA group was 23.6% lower than the model group. All these results shown in Fig. 10 indicate that the administration of CCl_4_ inhibited the expression of the genes regulated by Nrf2 including CAT, GPX2 and SOD3, but the treatment with GA could enhance the target genes expression to some extent.

### Effect of FeCl_2_-ascorbic Acid Stimulated Lipid Peroxidation and DPPH Radical Scavenging Activity

**Figure 11:**
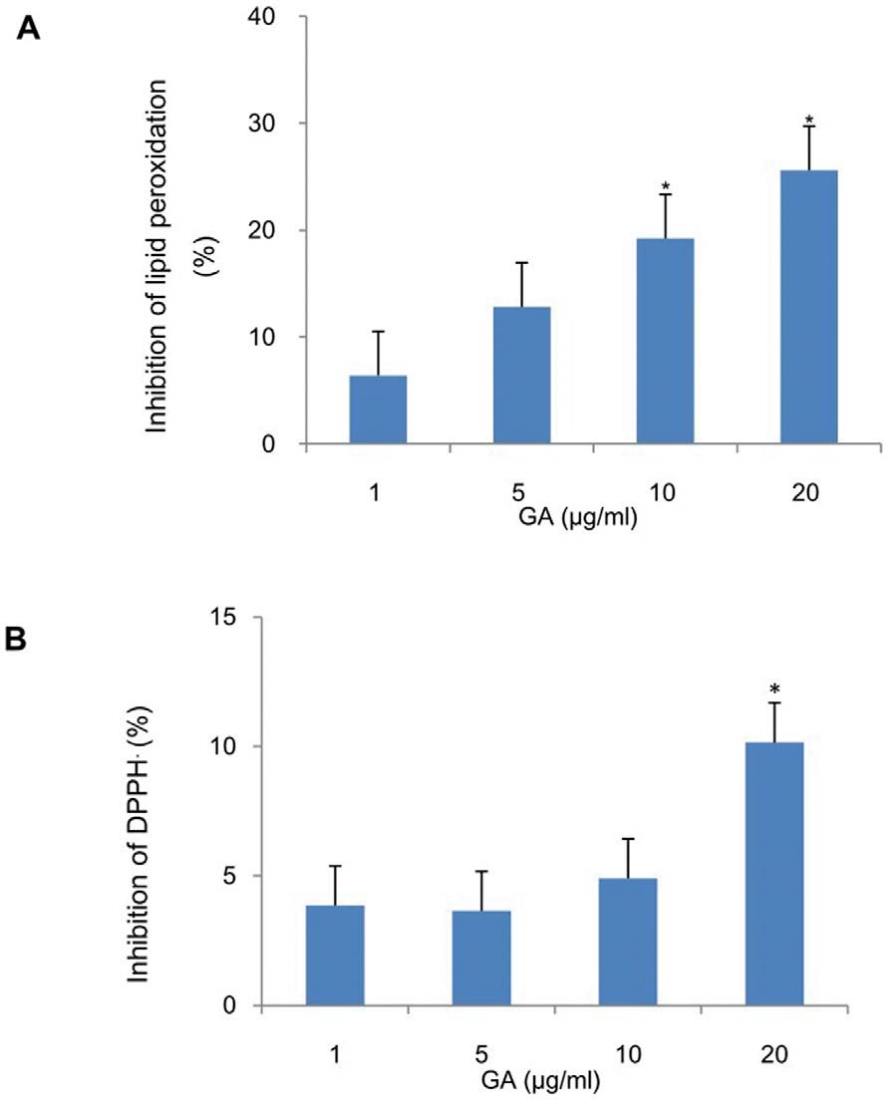
Inhibitory effects of GA on FeCl_2_-ascorbic acid stimulated lipid peroxidation and DPPH radical scavenging activity. (A) The mouse liver homogenates were stimulated with FeCl_2_-ascorbic acid in the presence or absence of GA. The lipid peroxidation was measured as described in Section 2. Control value of lipid peroxidation in liver homogenates. (B) The DPPH radical scavenging activity was determined by the DPPH assay in the presence or absence of GA and the scavenging activity was measured as described in Section 2. The value is presented as the mean of the percentage inhibition±SD for three independent experiments, performed in triplicate. *Significantly different from the control (GA = 0 µg/ml) at *p*<0.05.

The anti-lipid peroxidation and DPPH scavenging effects of GA were investigated in liver homogenates to determine the antioxidant effects of GA in terms of the mechanism of their anti-fibrosis effects. Consistent with the results CCl_4_-induced hepatic lipid peroxidation, GA showed a dose-dependent inhibition of the FeCl_2_-ascorbic acid stimulated lipid peroxidation in the liver homogenate (Fig. 11A) and also exhibited DPPH radical scavenging activity in a dose-dependent manner (Fig. 11B). Taken together, GA has antioxidant activity *in vitro.*

## Discussion

Chronic liver diseases constitute a global connection, and the medical treatments for these diseases are usually difficult to handle and have limited efficacy. Therefore, it is necessary and considerable interest to find new medicines for the treatment of liver diseases [35]. For that reason, our experiment is interesting.

In an attempt to model the liver fibrosis process, CCl_4_ has been used to experimentally induce liver injury in rodents widely. A single dose of CCl_4_ leads to centrizonal necrosis and steatosis [38], while prolonged administration CCl_4_ leads to liver fibrosis. It is said that the liver fibrosis model induced by CCl_4_ is the best-characterized system among the xenobiotic-induced liver damage [39]–[44]. It is now generally accepted that the liver fibrosis is induced by oxidative stress, which was produced by CCl_4_ *in vivo*. As reported, the liver fibrosis induced by CCl_4_ is the result of reductive dehalogenation. CCl_4_ *in vivo* is catalyzed by CYP450, then it forms the highly reactive trichloromethyl free radical (CCl_3_•). This radical readily interacts with O_2_ to form a more reactive trichloromethylperoxy radical (CCl_3_OO•), which is capable of binding to protein or lipid, or of abstracting a hydrogen atom forms an unsaturated lipid, which leads to lipid peroxidation and liver damage as well as plays significant roles in the pathogenesis of liver fibrosis [39]–[43], [45]–[52].

GA has been widely used for the treatment of liver diseases, such as chronic hepatitis C. It has been reported that GA can treat chronic hepatitis C by inhibiting the type I collagen gene transcription [53]. It can treat liver fibrosis by decreasing the collagen deposition and downregulation of the type I procollagen as well as the alpha-SMA on RNA expression [54]. But in our study, we found that GA can inhibit the free radical toxicity and lipid peroxidation *in vitro*, which is close to itself’ s antioxidative ability. Moreover, our study showed that GA has an inhibitory effect on liver fibrosis *in vivo,* which is probably associated with its activation of antioxidant protein that was regulated by Nrf2. All these results referred to front are consistent with the others researchers’ opinion on GA [55]–[57]. It has showed that, the increased serum levels of GOT and GPT have been attributed to the damaged structural integrity of the liver, because these are cytoplasm in location and released into circulation after cellular damage [41], [42]. And both of the increased levels of serum MAO and the homogenate MAO are the standards of liver fibrosis [58], [59]. Upon our study, liver fibrosis induced by CCl_4_ intoxication in mice was established from significant alterations in the serum GOT, GPT and MAO levels as found by previous researchers. However, the serum GOT, GPT and MAO activities declined in the GA-supplemented groups, suggesting that GA protected the mice against CCl_4_-induced liver fibrosis to some extent. During the different dose of GA administration, the 50 mg/kg GA and 25 mg/kg GA decreased these in serum and homogenate levels mostly. In parallel with the alteration of liver function markers, these phenomena were also confirmed by histological observation (Fig. 1; Fig. 2; Fig. 4; Fig. 5; Fig. 6; Fig. 7). In our study, GA can relieve the inflammatory cell infiltration, hydropic degeneration and fibrosis around the central vein as well as focal necrosis to some extent, which is induced by the administration of CCl_4_. Then the improvement of enzyme level to a normal value indicated that GA could treat liver fibrosis to some extent.

Oxidative stress is a key factor on liver fibrosis. Extensive studies with model systems, and with biological material *in vitro*, have clearly showed that free radicals which are produced along with oxidative stress, can produced chemical damage to lipids, proteins, and carbohydrates [60]. So if such free radicals produced in liver, it can make great degeneration disease-liver fibrosis. It can be restrained by endogenous free radical scavengers such as SOD, CAT and GSH-Px. SOD reduces the concentration of highly reactive superoxide radical by converting it to H_2_O_2_ whereas CAT and GSH-Px decomposes H_2_O_2_ and protect the tissues from highly reactive hydroxyl radicals [42], [43]. And our study showed that, the treatment of GA can reverse the toxic effects of CCl_4_ by restoring the activities of antioxidant enzymes towards the level of control animals. It seems likely that CCl_4_ administration cause oxidative stress in liver *via* the generation of free radicals whereas GA relieves the liver injuries by upregulating the activity of CAT, SOD and GSH-Px to scavenge of free radicals.

As reported, the lipid peroxidation is one of the major outcomes of free radical-mediated injury that directly damages membranes and generates a number of secondary products *in vivo*, both from fission and endo-cyclization of oxygenated fatty acids possessing toxic activities. It is close to liver fibrosis. It is not only as the standard of the liver fibrosis, but could also directly induce liver fibrosis [61], [62]. And it is said that one of the principle causes of CCl_4_-induced liver fibrosis is lipid peroxidation of hepatocyte membranes by free radical derivatives of CCl_4_ [61], [62]. MDA, an end product of membrane lipid peroxidation, is one of the most widely used indicators for free radical mediated damage [63]. The observation of elevated levels of hepatic MDA in Group II (administered CCl_4_ alone) in our study is higher than that in any other groups. GA can decrease the high level of MDA induced by CCl_4_, which had made great interest since it provides additional evidence to show us an anti-liver fibrosis role of GA. That is consistent with the hypothesis that GA could reverse CCl_4_-induced liver fibrosis in mice by inhibiting the lipid peroxidation.

But either inhibiting lipid peroxidation or upregulating the antioxidatiant enzymes activity *in vivo* was regulated by the NF-E2-related factor 2 (Nrf2). Nrf2, a structure of leucime zipper, is highly expressed on detoxification organs, such as liver and kidney [64]. Under normal conditions, Nrf2 is located in the cytoplasm where it forms an inactive complex with its repressor Kelch-like ECH2-associated protein [65]. Upon cell stimulation, Nrf2 dissociates from Keap 1, translocates into the nucleus where it binds to ARE, promotes the expression of Nrf2 target genes, and then increases the effect of antioxidative enzymes, such as CAT, SOD as well as GSH-Px [66]–[71], [73]. Therefore, upregulation of Nrf2 in nuclear can result in a reduction in the level of the reactive metabolites, and correspondingly, less tissue injury. In our study, immune blot analysis showed that the mice receiving CCl_4_ alone showed a dramatic down-regulation in Nrf2 protein level in both nuclear and cytoplasm which was reversed by GA treatment (Fig. 8 and Fig. 9). And it is showed that the level of Nrf2 expression is a good correlation with the treatment of GA against CCl_4_ induced liver fibrosis in mice. These results showed that the enhanced expression of Nrf2 in the nuclear transplantation by GA is consistent with the increase activities of antioxidant enzymes by GA *in vivo*. But the mechanisms of Nrf2 up-regulation by GA require further investigation. In this study, we hypothesized that the activation and upregulation of Nrf2 enhancing the target genes’ expression in mice plays an important role in enhancing antioxidative activity against liver fibrosis induced by CCl_4_ [73]–[74].

Gene’s expression studies are useful supplements to protein examinations, as the mRNA levels represent a snapshot of the cell activity at a given time. Nrf2 is also known to act as a mediator in CCl_4_-induced liver fibrosis by regulating relative gene of antioxidant enzymes expression. And in our study, SOD3 mRNA, CAT mRNA and GPX2 mRNA were chosen as the target genes of Nrf2 in protecting liver from fibrosis [16]–[18], [73], [74]. The SODs represent the major cellular defense system against superoxide radicals. In mammalian tissue, three isoforms of SODs have been identified: the cytoplasmic CuZnSOD (SOD1), the mitochondrial MnSOD (SOD2) and the extracellular SOD (EC-SOD or SOD3). There is reported that SOD1 and SOD2 genes didn’t decrease by the oxidative stress but the SOD3 gene, which is consistent with our study [75]. SOD3 is mainly secreted into the extracellular space, but a smaller proportion is also found in plasma and other extracellular fluids. It is the least studied enzyme, but recent data supports an important role for SOD3 in maintaining oxidative homeostasis in extracellular matrix and in nucleus as well [76]–[78]. And the disruption of the SOD3 gene in mice does not produce obvious pathologies under normal conditions, but these mice are more prone to environmental stressors. Moreover, very little is known about the regulation and potential of SOD3 in the prevention of CCl_4_-induced liver fibrosis. And there is a presence of an antioxidant responsive element (ARE) to Nrf2, which is the master regulator of the antioxidant response binds has been reported in 5′-untranslated region of SOD3 gene. This region is only in SOD3 promoter and not in SOD2 promoter region. [75], [79]. In our study, we have reported that GA can enhance the SOD3 gene expression, which is regulated by Nrf2 in the prevention of CCl_4_-induced liver fibrosis. This is the first direct evidence for the role of SOD3 in antioxidant-mediated prevention liver fibrosis and regulation of SOD3 through Nrf2 after the treatment of GA. Catalase is a 240,000 molecular weight tetrameric hemoprotein, which is located mainly in the peroxisomes, has reductive activity mainly for small molecules rather than lipid hydroperoxide products of lipid peroxidation [80]. CAT catalyzes the reduction of H_2_O_2_ to water (H_2_O) and oxygen (O_2_) under the regulation of Nrf2 [81]. During our study, CAT enzymes’ activity is consistent with the expression of CAT mRNA in livers. Hence, GA protects liver from highly reactive hydroxyl radical (•OH) derived from H_2_O_2_ by upregulating Nrf2, enhancing its target gene-CAT expression and increasing the activity of CAT enzyme. GPXs, a family of selenium-dependent glutathione peroxides, contain five members. GPX2, which is responsible for glutathione-dependent hydrogen peroxide-reducing activity in the epithelium of the gastrointestinal tract, was first described in 1993 as a novel isoenzyme exclusively expressed in the gastrointestinal tract. GPX2 is the same with other glutathione peroxidases in some function. It reduces fatty acid hydroperoxides and, due to its expression in the intestinal epithelium, has been suggested to function as a barrier against hydroperoxide absorption [82]–[85]. As reported, GPX2 gene as an unorthodox target for Nrf2 is regulated by Nrf2, which canonically responds to antioxidants [85]. Intrigued by this corollary, we analyzed whether the enhancing expression of GPX2 of GA is regulated by the Nrf2/Keap1 system and we can see that the expression of GPX2 can be inhibited by the regulated of Nrf2 in the livers administrated by CCl_4_. But GA can reverse it to some extent.

In addition, GA has an obvious antioxidative activity *in vitro. In vitro,* lipid peroxidation in liver homogenate can proceed in a nonenzymatic way. The process is induced by ascorbate in the presence of Fe^2+/3+^. Furthermore, it has been reported that, Fe^2+^ and ascorbic acid stimulated lipid peroxidation in rat liver microsome and mitochondria [36]. *In vitro* lipid peroxidation experiments were carried out to clarify the mode of GA action. According to the results obtained, GA inhibited FeCl_2_-ascorbic acid-stimulated lipid peroxidation in liver homogenate and made it presence of a dose-dependence (Fig. 11A). And these results showed that GA exercised powerful free radical scavenging activity on the DPPH free radical generated using the DPPH• assay (Fig. 11B), and acted by scavenging free radicals and oxygen species formed during the CCl_4_ metabolism. These results are consistent with the previous reports showing that GA is an oxygen free radical scavenger. Active oxygen species and free radicals are the code epidemic factor of liver fibrosis. GA with antioxidant properties might contribute towards the partial or total alleviation of this damage [72].

**Figure 12:**
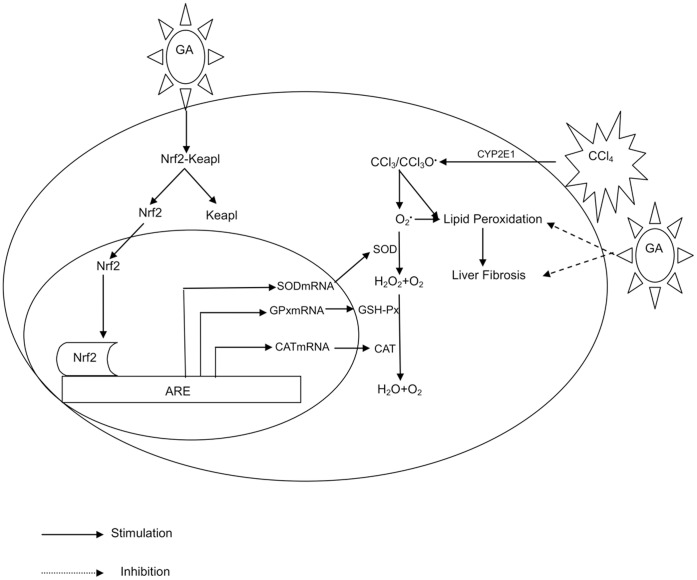
Potential mechanisms for anti-fibrosis mechanism of GA against CCl_4_-induced liver fibrosis in mice.

All together, the results in this study were summarized in the scheme of Fig. 12. It demonstrated that GA has hepatoprotective action upon CCl_4_-induced chronic liver fibrosis in mice. These results show that the treatment effect of GA may be due to its ability to promote Nrf2 nuclear transcription and enhance the Nrf2 target genes’ expression, then inducing the MDA content and oxidases (GOT/GPT/MAO) activity decreased, the anti-oxidases (SOD/CAT/GSH-Px) activity increased toward the formation of trichloromethyl radicals that are capable of inducing liver fibrosis.
